# Prostate Gland Lengths and Iceball Dimensions Predict Micturition Functional Outcome Following Salvage Prostate Cryotherapy in Men with Radiation Recurrent Prostate Cancer

**DOI:** 10.1371/journal.pone.0069243

**Published:** 2013-08-09

**Authors:** Imran Ahmad, Gabriela Kalna, Mohamed Ismail, Fiona Birrell, Sue Asterling, Elaine McCartney, Damien Greene, John Davies, Hing Y. Leung

**Affiliations:** 1 Urology Group, Beatson Institute for Cancer Research, Bearsden, Glasgow, United Kingdom; 2 Department of Urology, National Health Service Greater Glasgow and Clyde, Glasgow, United Kingdom; 3 Department of Urology, The Royal Surrey County Hospital and St Luke's Cancer Centre, Guildford, Surrey, United Kingdom; 4 Department of Urology, Sunderland Royal Hospital, Sunderland, United Kingdom; 5 Clinical Trials Unit, Beatson West of Scotland Cancer Centre, Glasgow, United Kingdom; Innsbruck Medical University, Austria

## Abstract

**Introduction:**

Tissue cryoablation is a potential curative option for solid malignancies, including radiation recurrent prostate cancer (RRPC). Case series of salvage cryotherapy (SCT) in RRPC have reported promising disease free survival (DFS) outcomes and acceptable toxicity profile. While many men receive SCT, no predictive factors for treatment induced side effects are known. The aim of this study is to validate the oncologic outcome of SCT in a large multi-centre patient cohort and to identify potential parameters associated with an increased risk of micturition symptoms.

**Patients and Methods:**

In this retrospective analysis, we studied 283 consecutive patients with RRPC treated by SCT in three independent U.K. centres (between 2001 and 2011). Two freeze-thaw cycles of transperineal cryotherapy were performed under transrectal ultrasound guidance by a single surgeon in each of the 3 sites. We analysed clinico-pathological factors against tumour response. Functional outcomes were assessed by continence status and IPSS questionnaire. Predictive factors for SCT-induced micturition symptoms were analysed in a sub-group (n = 42) of consecutive cases.

**Results:**

We found that nadir post-SCT PSA levels strongly associated with DFS. The DFS rates at 12- and 36-month were 84% and 67% for the ≤1 ng/ml group and 56% and 14% for the >1 ng/ml group, respectively (p<0.001). Correlative analysis revealed highly significant association between patients' post-SCT micturition status with prostate gland and iceball lengths following SCT. Finally, in a reduction model, both gland length and maximal length of iceball were highly associated with patients' IPSS outcome (p<0.001).

**Conclusion:**

We report the largest European patient cohort treated with SCT for RRPC. Oncologic outcome guided by nadir PSA of <1 ng/ml is consistent with earlier single-centre series. For the first time, we identified physical parameters to predict micturition symptoms following SCT. Our data will directly assist on-going and future trial design in cryotherapy in prostate cancer.

## Introduction

Recurrence following radiotherapy (RT) is a difficult treatment dilemma, which is not uncommon amongst prostate cancer (PC) patients. Over 30% of patients are reported to develop cancer recurrence following primary RT [1], with most of these patients managed by androgen deprivation therapy (ADT). Salvage radical prostatectomy (RP) is far more challenging than primary RP [2]–[5], with radiation induced vasculitis, fibrosis and tissue plane obliteration leading to significant risk of rectal injuries, anastomotic strictures and urinary incontinence while positive margin rates can be over 50% [2], [6]. Despite this, in the younger patient, salvage RP offers superior biochemical disease-free survival and may potentially offer the best chance of cure [7].

Taking this into consideration, local ablative treatments such as salvage cryotherapy (SCT) and high-intensity focused ultrasound (HIFU) are increasingly considered as viable options for second line therapies with intent for cure. However these minimally invasive procedures are not without potential risks of serious treatment related toxicities, including genito-urinary symptoms, erectile failure and less commonly rectal injury [8]. Overall, these risks are considered acceptable, with disease free survival (DFS) of up to 56% and 39% at 5-years and 10-years following SCT, respectively [8]–[11]. While treatment related toxicities are often reported, little is known about the relative risk factors among individual patients undergoing the procedure. Here, we reported our multi-centre case series and considered factors that directly impact on the risk of side effects as well as treatment efficacy.

## Materials and Methods

### Trial Registration

public.ukcrn.org.uk, CROP Trial, URL: StudyDetail.aspx&quest;StudyID&hairsp;&equals;&hairsp;10051.

### Patients, follow up and data collection

All patients who underwent SCT for radiation recurrent prostate cancer between 2001–2011 at the three participating U.K. centres were included, namely Glasgow (Gartnavel General Hospital), Sunderland (Sunderland Royal Hospital), and Guildford (the Royal Surrey County Hospital). Patients were selected based on evidence of primary RT failure and histologic evidence of localised PC in the absence of metastatic disease (negative MRI and isotopic bone scan).

Patients were initially followed up by PSA monitoring at 6 weeks, then 3 monthly intervals till 12 months, followed by 6 monthly, up to a maximum of 72 months. Data collected included patient age, PSA levels before SCT, clinical stage, biopsy Gleason score, follow up PSA levels and survival status as well as results from bone scan, CT and MRI. Recurrence was defined using the Phoenix definition of nadir plus 2 ng/ml as well as radiological, histological, or clinical evidence of recurrent prostate cancer. Disease free survival (DFS) was defined as the time in months from SCT to date of recurrence. Patient functional outcomes were assessed by the following parameters: continence status (use of ≤1 or ≥2 pads per day), IPSS total score of 7 symptoms (range 0–35) and IPSS quality of life score (range 1–6).

### Prostate cryoablation and data on physical parameters

SCT was performed as described previously [12], [13]. In brief, cryoablation of the prostate gland was performed using the Seednet^TM^/Presice^TM^ (GalilMedical, Plymouth Meeting, PA, USA) or the Cryocare CS system (Endocare, Inc., Irvine, CA, USA). Cryo-needles are inserted into three imaginary levels into the prostate; anterior, middle and posterior. Thermocouples are placed in critical positions to monitor the temperature of the anterior rectal wall and the adjacent structure including the sphincter. Each freeze cycle was started with the anterior needles; once the entire width of the prostate was covered anteriorly, the middle and then the posterior row of cryoneedles were switched on. Two cycles of freezing and thawing were performed to published consensus [14] with lowest temperature within the prostate to below −30°C to −40°C for 5–10 minutes. Real time assessment of the dimension and contour of iceballs was performed using transrectal ultrasonography in sagittal and coronal views.

A cohort of consecutive patients (n = 42) treated in Glasgow between 2008–2011 were analysed for relationship between physical parameters of cryoablation (temperature achieved and iceball length). Data were collected for (1) the lowest temperature obtained in each lobe of the prostate, (2) the longitudinal length of the iceball (parallel to the anterior rectal wall) at the end of each freeze cycle, and (3) the prostatic gland length.

### Statistics and correlative analysis

Chi-squared tests were used for grouped data and Student t tests for continuous data. Statistical analyses on clinico-pathologic correlations and disease free survival were performed using Prism v5.0c (GraphPad Software Inc., La Jolia, CA, USA). Univariate and multivariate generalized linear models (glm) with Poisson response were used to test effects of the physical parameters from cryoablation on post-SCT IPSS values, taking into consideration of the skewed distribution of positive discrete values of IPSS. For multiple IPSS values of individual cases, minimum, maximum and mean, rounded up to the nearest whole number, were considered in the analysis. First, full list of predictors was considered in a multivariate model. Then, using the automated model selection package ‘glmulti’ from R environment (http://www.r-project.org), all possible reduced model formulae were generated and the ‘best’ models, based on Akaike information criterion (AIC), were selected. Finally, we focused on model(s) that had a simple explanation and possible clinical application.

### Ethics

Prostate cryotherapy is a clinically valid treatment option for radiation recurrent prostate cancer. There are no ethical issues with the reported study.

## Results

### Analysis of treatment efficacy

**Figure 1:**
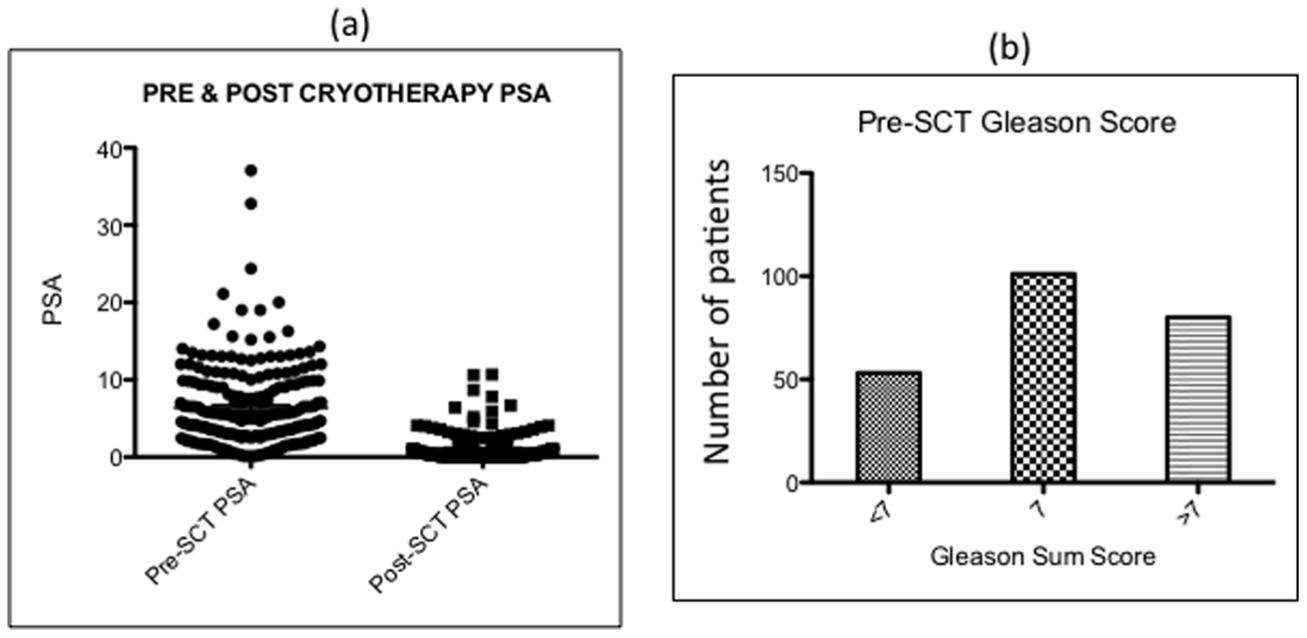
PSA and Gleason sum scores of SCT population. (a) Dot blot showing the pre and post-SCT nadir PSA for the entire patient cohort, (b) Bar chart representing the numbers of patients with different Gleason sum scores prior to SCT.

**Figure 2:**
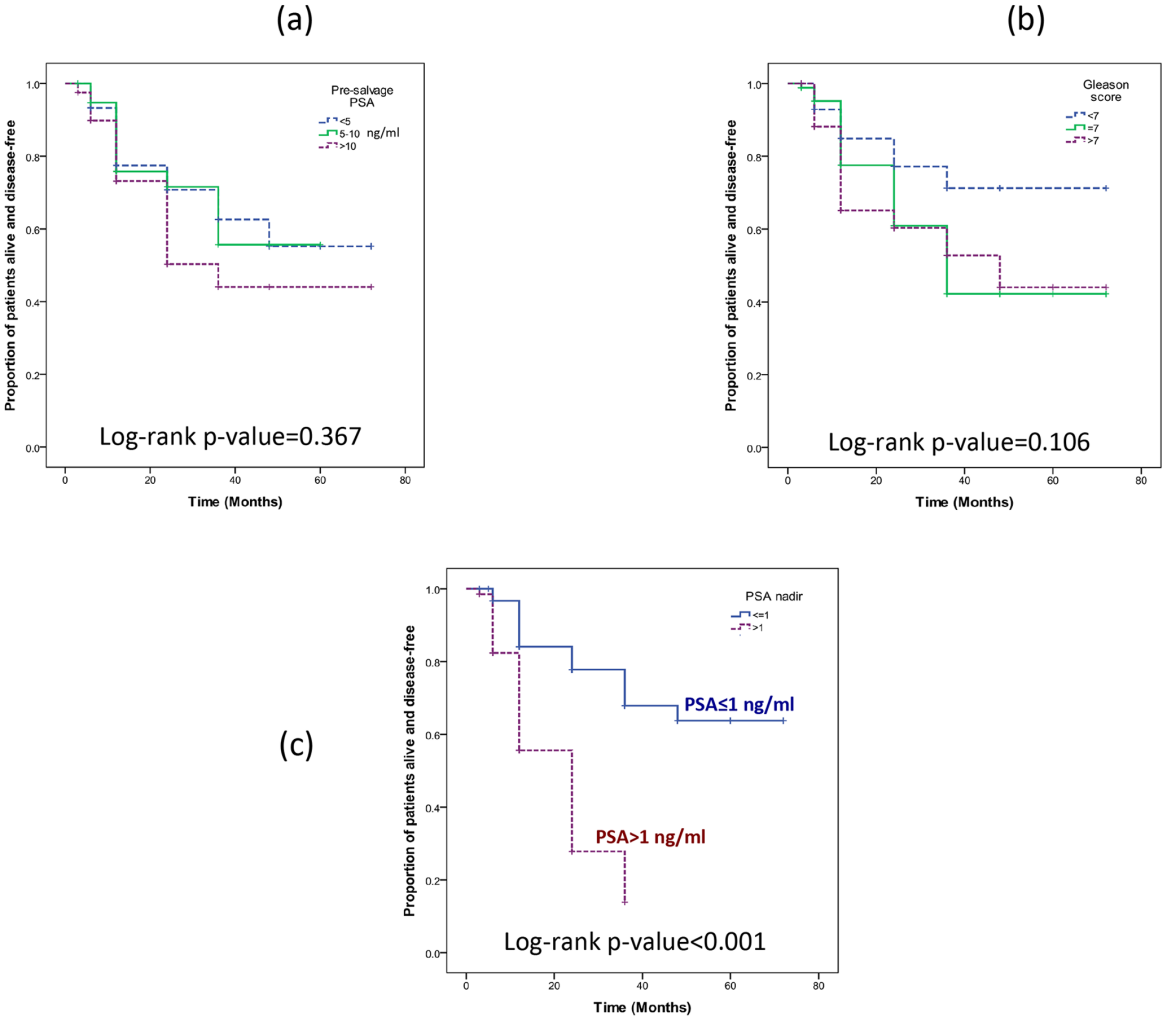
Disease free survival by (a) Pre-SCT PSA, (b) Gleason Score and (c) PSA nadir.

Pre-SCT demographic patient information (n = 283) is summarised in Tables 1 and S1. Patients with data on the three specific risk factors were included in the analysis, namely pre-SCT PSA (n = 197), pre-SCT Gleason score (n = 195) and post-SCT nadir PSA (n = 227) (Figures 1, S1, S2). Patients were followed up for an average of 23.9 months (IQR 3–36 months). Contrary to previous reports on SCT, our patients with a pre-SCT PSA of <5, 5–10 and >10 ng/ml had very similar 12-month disease free survival (DFS) rates, at 78%, 76% and 73% respectively [10]. At 5-year, patients with PSA up to 10 ng/ml had DFS rates of ∼55%, while those with PSA >10 ng/ml had DFS rate slightly lower at 44% (not statistically different: p = 0.367, log rank test; Figure 2a and Table S2). A similar analysis of Gleason score showed some variability in the DFS rates for patients with scores of <7,  = 7 and >7, with 12-month DFS rates at 85%, 76% and 65%, respectively, and 5-year DFS rates at 71%, 42% and 44%, respectively. A log rank test indicated that the hazard functions for these three groups were not statistically different at the 5% significance level (p = 0.106; Figure 2b and Table S2).

**Table 1:** Summary demographics of our series of patients who received SCT.

Age at time of Rx	Number of Patients
	(Percentage in bracket)
≤65	130 (45.5%)
>65	153 (54.5%)
**Pre Rx Gleason**	
<7	53 (21.4%)
7	108 (43.5%)
>7	87 (35.1%)
**Pre Rx PSA, ng/dl**	
<5	120 (49.2%)
05-Oct	74 (30.3%)
>10	50 (20.5%)
**Clinical T Score**	
T1	20 (9.3%)
T2	134 (62.0%)
T3/4	62 (28.7%)

Breakdown of patient demographics and tumour parameters from the three cryotherapy centres can be found in Table S1. Please note that not all categories add up to 283, since data was incomplete for some patients as regards Pre Rx Gleason, Pre Rx PSA and Clinical T score. (Rx  =  cryotherapy).

Consistent with a recent single centre report [10], the DFS rates in our patient cohort were significantly different at the 5% significance level when patients were stratified according to their post-SCT nadir PSA levels into ≤1 and >1 ng/ml groups. The 12-month and 36-month DFS rates were 84% and 67% for the ≤1 ng/ml group and 56% and 14% for the >1 ng/ml group respectively. A log rank test indicated that the hazard functions were statistically different at the 5% significance level (p<0.001) (Figure 2c and Table S2).

### Functional outcome and correlative analysis of cryotherapy parameters

Functional outcomes of our series are comparable to previously published literature, with incontinence rates up to 36% and fistula rates up to 2.6% being reported [9], [15]–[17]. We observed long-term incontinence rates of 12% (using >1 pad per day beyond 6 months post-SCT) and fistula rate of 1.8%, highlighting the acceptability of this salvage modality. Our erectile dysfunction (ED) rate was at 83%, although at least half of these patients had some form of pre-existing ED, Table 2. We observed a statistically significant change in the international prostate symptom score (IPSS), with scores increasing (or symptoms worsening) after SCT (Paired t-test, p<0.0001) (Figure S3). A total of 23 patients out of the 283 (8.1%) treated patients required transurethral resection of the prostate for obstructive urinary symptoms.

**Table 2:** Post-SCT complications and the number of patients affected by each complication.

Complication	Number	%
Urinary Incontinence	37	12
Erectile Dysfunction	246	83
Lower Urinary Tract Symptoms	42	14
Prolonged Perineal Pain	11	4
Urinary Retention	20	7
Recto-Urethral Fistula	5	1.8

We hypothesised that treatment related toxicities such as incontinence following SCT directly relate to the dimensions of prostate gland and iceball generated. Therefore, we carried out statistical modeling to investigate for correlation between physical parameters (temperature and iceball dimensions) and patient functional status (incontinence and micturition functional scores as assessed by IPSS) in a cohort of 42 consecutive patients. Full set of data on prostate gland lengths and physical parameters from cryotherapy procedures were available on 31 patients, including minimal temperatures within the prostate achieved and iceball lengths at the end of each freeze cycles. Multivariate analysis of these parameters revealed highly significant association between patients' post-SCT IPSS (both mean and maximal) scores with gland and iceball lengths at the end of first and second cycle of freezing (Table 3). The absolute minimal temperatures recorded within the prostate were interestingly not associated with IPSS scores. We carried out additional multivariate analysis of the best reduced-model from the ‘glmulti’ selection. Table 4 demonstrates a highly significant association between gland length and ice length following the second freeze cycle. To develop a simple platform for correlative analysis in future studies, IPSS scores were analysed against a single minimal temperature and the maximal iceball length for each cryotherapy procedure as well as the respective gland length. We found gland length and maximal iceball length to be highly associated with individual patients' IPSS outcome (Table 5). It is likely that the relative lengths of individual prostate length and the respective iceball size may determine the actual risk to the surrounding tissue. We therefore examined if the ratio of the iceball dimension and the prostate length is related to patient outcome. While not reaching statistical significance, a trend was observed. Two of the twenty (10%) cases with ratio of MaxIceball/gland below 1.2 has IPSS >20 while 5 out of 11 cases (45%) with ratio above 1.2 has IPSS >20. Finally, we found no evidence of association between patient continence status and prostatic temperature or iceball length.

**Table 3:** Summary of multivariate analysis.

Parameters	Mean	Std. Dev.	n	Multivariate (Mean IPSS); p values	Multivariate (Max IPSS); p values
**Right_Lobe_1_C**	43.6	14.24	35	0.45094	0.27733
**Right_Lobe_2_C**	39.9714	12.8761	35	0.999	0.700769
**Left_Lobe_1_C**	38.6286	17.8162	35	0.68923	0.327031
**Left_Lobe_2_C**	33.8529	12.702	34	0.34296	0.108465
**Gland_Length**	32.6857	3.80226	35	***4.35E-05***	***3.20E-06***
**Iceball_Length_ 1_C**	37	2.72915	30	0.17239	0.060049
**Iceball_Length_ 2_C**	36.9677	3.58221	31	***0.00178***	***0.000642***

Multivariate analysis of SCT parameters reveals highly significant association between dimensions of gland length and the iceball with patients' IPSS scores. (1_C and 2_C denotes 1^st^ and 2^nd^ cycles, respectively; Right and left lobe signified the nadir temperature recorded below 0°C; Iceball Length signifies the dimension of iceball parallel to the anterior rectal wall, measured in mm, at the end of each freeze cycle; n  =  patient numbers).

**Table 4:** Simplified analysis of gland length and the largest iceball dimension against IPSS scores.

Parameters	Mean	Std. Dev.	n	Multivariate (Mean IPSS); p values	Multivariate (Max IPSS); p values
**Gland_Length**	32.6857	3.80226	35	***0.02417***	***0.00645***
**Iceball_Length_ 1_C**	37	2.72915	30	0.47602	0.86751
**Iceball_Length_ 2_C**	36.9677	3.58221	31	***0.00604***	***0.00284***

Significant association between gland length and iceball length with (mean and maximal) IPSS scores were observed. (1_C and 2_C denotes 1^st^ and 2^nd^ cycles, respectively; n  =  patient numbers).

**Table 5:** Association between gland length and maximal length of iceball recorded with patient IPSS outcome.

Parameters	Mean	Std. Dev.	n	Multivariate (Mean IPSS); p values	Multivariate (Max IPSS); p values
**MaxLobe**	50.4857	14.6516	35	0.06555	0.05956
**Gland_Length**	32.6857	3.80226	35	***0.00904***	***7.24E-03***
**MaxIceball**	37.9063	2.93323	32	***8.27E-05***	***8.72E-06***

(Max Lobe represents the lowest temperature below 0°C recorded in the prostate; Max Iceball is taken as the longest dimension of iceball recorded for the entire procedure; n  =  patient numbers.).

## Discussion

The published literature on the incidence of genitourinary toxicity following SCT is highly variable. The underlying basis for such widely varied functional outcome has not been adequately considered. Here, we report the largest European patient cohort treated with SCT for radiation recurrent prostate cancer (RRPC). It is worth noting that patients who received androgen ablation in conjunction with SCT were excluded from this study. Our toxicity outcome data (Table 2) are in keeping with reported literatures, further supporting SCT as an acceptable treatment for radiation recurrent PC. To our knowledge, this is the first study successfully attempted to identify potential means to objectively assess and predict the risk of treatment toxicity following local cryoablative treatment in any organs. This will impact the applications of cryotherapy to different tumour types in both primary and salvage treatment settings. We collected physical parameters during SCT procedures and tested for their association with symptomatology related to micturition function. We found significant associations between dimensions of the prostate gland and iceball on one hand and IPSS scores on the other hand (Table 3–5). Interestingly, the absolute minimal temperatures recorded within the prostate did not correlate with IPSS scores. Conceptually, it is clearly logical that the relative prostate gland size and iceball dimension may correlate with functional outcome, with the sphincteric muscle being at high risk of freeze injury if the iceball expands beyond the physical dimension of the prostate gland during cryotherapy. Our findings will impact on future refinement of SCT techniques in ensuring optimal treatment efficacy while minimising toxicities due to freezing injury to adjacent tissue. The approach for analysis presented in this report provides a simple but objective methodology to prospectively assess individuals' risk in experiencing significant urinary symptoms and should be incorporated in prospective cryotherapy studies [18], [19]. Though not reaching statistical significance, based on our data, an iceball extending significantly beyond the prostatic gland length (ratio of >1.2 fold) may result in higher risk of micturition-related toxicities. Our current working model is that patients with glandular size such as between 20–30 cm^3^ are ideal for total prostatic cryoablation. Our reasoning is that, when treating patients with a moderate prostatic dimension, the cryotherapy practitioners may have the best chance in achieving therapeutic level of freezing while maintaining a ratio between iceball to prostate lengths to below 1.2. This hypothesis requires further investigation in future (prospective) studies.

Although consecutive patients were included in this multi-institute study, an important limiting factor is its retrospective nature, with heterogeneous within the patient populations [10], [20]. The impact of nadir PSA as an indicator of treatment response is consistent with other recent publications. Those patients with a high PSA nadir following SCT (>1 ng/ml and ≥0.6 ng/ml respectively) are at increased risk of recurrence [10], [21]. In addition, we also demonstrated significant treatment benefits with proper patient selection. Similar to report by Williams *et al*, we also highlight the role of pre-SCT biopsy and its prediction of disease recurrence [10]. Although a proportion were not included because of post RT changes making Gleason grading impossible, we found a trend of increased risk of disease recurrence following SCT in patients with tumour Gleason score ≥7. In patients who have had prior RT, the role of Gleason grade remains controversial. Currently, the American Society for Therapeutic Radiology and Oncology does not recommend routine prostate biopsy, but rather recommends following PSA levels as a marker for recurrent disease [22]. Finally, we were not able to study for the relationship between the schedules of previous radiation therapy and cryotherapy-related toxicities. It will be important to prospectively collect data on primary radiotherapy as any radiation-induced injuries are likely to influence tissue response to freezing.

## Conclusion

Our data from the largest European series of SCT for radiation recurrent prostate cancer to date demonstrated acceptable treatment efficacy and comparable treatment toxicities to published reports. Importantly, we showed that both the dimensions of the prostate gland length and the iceball size were significantly associated with patient IPSS scores.

## Supporting Information

Figure S1 **Data on pre and post-SCT nadir PSA levels for each of the three participating centres.** (GLA – Glasgow, SUN – Sunderland, GUIL – Guildford.).
(TIF)

Figure S2 **Gleason scores of radiation recurrent prostate cancer prior to SCT are shown for each the three centres.** (GLA – Glasgow, SUN – Sunderland, GUIL – Guildford.).
(TIF)

Figure S3 **Pre and (3-month) post-SCT IPSS levels for the Glasgow cohort.**
(TIF)

Table S1 **Summary demographics from the three participating centres.**
(TIF)

Table S2 **Breakdown of patient numbers for individual survival analysis in** **Figure 2** **.**
(TIF)
